# Duration of lithium treatment is a risk factor for reduced glomerular function: a cross-sectional study

**DOI:** 10.1186/1741-7015-11-33

**Published:** 2013-02-11

**Authors:** Alberto Bocchetta, Raffaella Ardau, Paolo Carta, Franca Ligas, Claudia Sardu, Antonello Pani, Maria Del Zompo

**Affiliations:** 1 Section of Neuroscience and Clinical Pharmacology, Department of Biomedical Sciences, University of Cagliari, Via Ospedale 54, Cagliari, 09124 Italy; 2 Unit of Clinical Pharmacology, Azienda Ospedaliero-Universitaria di Cagliari, Via Ospedale 54, Cagliari, 09124 Italy; 3 Department of Public Health, Clinical and Molecular Medicine, University of Cagliari, Cittadella Universitaria, Strada Provinciale Monserrato-Sestu Km 0.7, Monserrato, 09042 Italy; 4 Nephrology, Dialysis and Transplantation Unit, 'Giuseppe Brotzu' Hospital, Piazzale Ricchi 1, Cagliari, 09134 Italy

**Keywords:** lithium treatment, glomerular filtration, chronic kidney disease

## Abstract

**Background:**

The adverse renal effects of lithium have long been known, but glomerular insufficiency had been considered an unlikely event until recently, when new studies have raised concern regarding very long-term treatment. In this cross-sectional study, we examined glomerular function in a cohort of patients treated with lithium for up to 33 years and a control group of lithium-naïve patients treated with other mood-stabilizers.

**Methods:**

Patients with a diagnosis of recurrent or persistent affective disorders, examined between 1 October 2007 and 31 December 2009, were screened. Demographic and clinical data were extracted from clinical charts regarding two study groups: one for patients treated with lithium for at least 12 months and the other for patients never exposed to lithium. Multivariate regression analysis was applied: the dependent variable was the estimated glomerular filtration rate (eGFR) calculated from the last available serum creatinine value using the Modification of Diet in Renal Disease Study Group equation; the following independent variables, potentially associated with renal dysfunction, were included: gender, current age, duration of lithium treatment, cigarette smoking, hypertension, diabetes and dyslipidemia.

**Results:**

eGFRs lower than 60 ml/min were significantly more frequent in the group treated with lithium (38/139 = 27.3%) compared to lithium-naïve patients (4/70 = 5.7%) (*P *= 0.0002; Fisher's test). Regression analysis showed a significant effect on eGFR of age, gender and duration of lithium treatment but no effect of cigarette smoking, hypertension, diabetes or dyslipidemia. eGFR was estimated to decrease by 0.64 ml/min (95% confidence interval = 0.38 to 0.90; *P *= 0.00) for each year of lithium treatment.

**Conclusions:**

The duration of lithium treatment is a risk factor for glomerular failure, in addition to advancing age. For example, all patients aged 60 years or older may be estimated to undergo Stage 3 or more severe chronic kidney disease (namely an eGFR less than 60 ml/min) if treated with lithium for 30 years. These data may be added to the current debate on the balance between the protective effects of lithium on recurrent affective disorders and suicide and the risk of renal disease.

See related commentary article here http://www.biomedcentral.com/1741-7015/11/34

## Background

Lithium is the most effective long-term therapy for bipolar disorder [1,2] and is also effective in unipolar depression [3]. However, there has been concern about its safety [4]. In particular, adverse renal effects have long been known, varying from very frequent reversible polyuria [5] to irreversible kidney damage [6,7].

A review of early studies updated to the 1970s concluded that lithium nephropathy usually includes structural tubular damage with interstitial fibrosis but that glomerular damage is absent or minimal [8]. Based on the small number of reported cases, severe glomerular insufficiency and end-stage renal disease (ESRD) were considered unlikely events according to a review updated to the 1990s [9].

On the contrary, new studies have raised concern regarding glomerular effects, especially in patients treated for many years [10]. Therefore, debate has recently been revived [4]. In particular, it has been suggested that the risk of renal failure is to be weighed against the benefits obtained [11]. The latter include protection not only from recurrence of affective disorders but also from their otherwise high mortality, principally due to suicide [12].

As we are in possession of detailed clinical and laboratory data regarding patients on maintenance lithium treatment for up to more than 30 years, we were prompted to investigate their glomerular function with particular regard to the duration of treatment. In this cross-sectional study, we sought to identify any demographic or clinical variable potentially related to renal insufficiency in a cohort of patients treated with lithium for at least 12 months and in a control group of patients with similar psychiatric diagnoses treated with other mood-stabilizers.

## Methods

All consecutive patients with a diagnosis of recurrent or persistent affective disorders (International Classification of Diseases, 10th edition (ICD 10) F31, F33, and F34), examined between 1 October 2007 and 31 December 2009 at the Unit of Clinical Pharmacology, University Hospital, Cagliari, were screened.

The study protocol was approved by the local ethics committee of the University Hospital Cagliari, Italy (reference number 16/09/CE). All patients gave written informed consent before inclusion in the study. The study was performed in accordance with the Declaration of Helsinki.

Two study groups were selected based on the following criteria: one consisted of patients treated for at least 12 months with lithium (n = 139) and the other of patients never exposed to lithium (n = 70; treated with carbamazepine, valproate and/or lamotrigine).

The following data, potentially associated with the risk of renal dysfunction, were extracted from clinical charts: gender, age, duration of lithium treatment, current smoking habit (20 or more cigarettes daily), hypertension (currently treated with any antihypertensive medication), diabetes (currently treated with insulin or oral antihyperglycemic agents) and dyslipidemia (currently treated with any antilipidemic agent). In addition, the last available serum creatinine concentration was taken from the panel of laboratory tests requested on an annual basis. Serum creatinine concentration prior to the onset of lithium treatment was also extracted if available.

The traditional standardization method for serum creatinine was used. Estimated glomerular filtration rate (eGFR) was calculated from serum creatinine values using the equation proposed by the Modification of Diet in Renal Disease (MDRD) Study Group [13,14] with the '186' correction factor which also takes into account age, gender and ethnicity. To estimate the proportion of cases with different degrees of reduced glomerular function, patients were classified according to the following eGFR values: > 89 ml/min (normal kidney function), 60 to 89 ml/min (mildly reduced kidney function) and < 60 ml/min (chronic kidney disease (CKD) stages 3 to 5).

Demographic and other clinical variables collected for the subset of patients treated with lithium were compared with those from the subset of patients treated with other mood stabilizers, using simple descriptive statistics. Categorical variables were compared using χ^2 ^and continuous variables using independent sample *t*-tests. Difference was considered statistically significant if the *P *value was less than 0.05.

Multivariate regression analysis was applied with eGFR as the dependent variable and including the following independent variables: gender, current age, exposure or lack of exposure to lithium, duration of lithium treatment, cigarette smoking, hypertension, diabetes and dyslipidemia. Duration of lithium treatment regarding control patients was included in the overall regression analysis with the value of zero.

## Results

**Figure 1:**
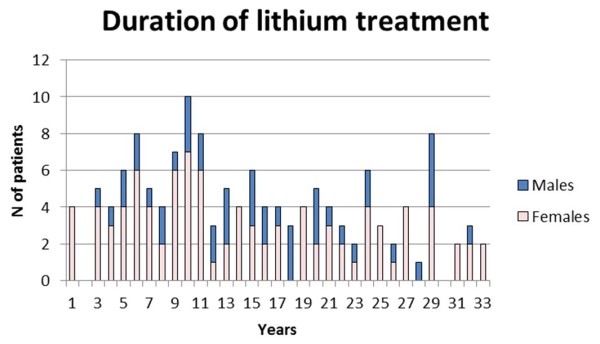
**Distribution of the duration of lithium treatment**.

Demographic and clinical characteristics of patients treated or not treated with lithium are shown in Table 1. The two subgroups did not differ with regard to gender ratio, psychiatric diagnoses or presence of the nephropathy risk factors being considered (cigarette smoking, hypertension, diabetes and dyslipidemia). Patients on lithium, when last evaluated, were older than controls treated with other mood-stabilizers (Table 1). The distribution of the duration of lithium treatment is shown in Figure 1.

**Table 1:** Demographic and clinical characteristics of patients treated or not treated with lithium.

Demographic and clinical characteristics	Patients treated with lithium	Lithium-naïve patients	*P*
	Number = 139	Number = 70	
Male: number (%)	44 (31.7)	17 (24.3)	n.s.
Mean current age in years (SD)	58.0 (14.0)	50.0 (12.4)	0.0001
Bipolar disorder (ICD10 F31): number (%)	119 (85.6)	58 (82.8)	n.s.
Other affective disorders (ICD10 F33-34): number (%)	20 (14.4)	12 (17.2)	n.s.
Cigarette smoking: number (%)	35 (25.2)	11 (15.7)	n.s.
Hypertension: number (%)	40 (28.8)	14 (20.0)	n.s.
Diabetes: number (%)	16 (11.5)	8 (11.4)	n.s.
Dyslipidemia: number (%)	76 (54.7)	34 (48.6)	n.s.

ICD10, International Classification of Diseases, 10th edition; n.s. non-significant; SD, standard deviation.

With regard to eGFR classes, the distribution between patients on lithium and patients never exposed to it differed significantly (data not shown). In particular, lithium patients appear to shift towards the lower ranges: for example, eGFR lower than 60 ml/min, namely Stage 3 or more severe CKD stages, was significantly more frequent in the lithium group compared to the patients who never received lithium (38/139 = 27.3% versus 4/70 = 5.7%) (*P *= 0.0002; Fisher's test). However, the age difference (Table 1) might in part explain the different distribution in eGFR classes. It was not possible to weigh the groups further, primarily because older patients with bipolar spectrum disorders treated at our clinic have little chance of never having been exposed to lithium. Indeed, eGFR distribution did not differ between controls and patients who were about to start lithium (a mean of approximately 13 years prior to the current evaluation) (data not shown).

In addition to the age difference between groups, the limitations of this study, principally due to its cross-sectional design, include: a) lack of detailed data regarding hypertension, diabetes, and dyslipidemia (duration and type of medication taken over the years) and/or presence of other cardiovascular diseases; and b) lack of detailed data regarding cigarette smoking (for example, an estimate of exposure in terms of pack-years). Moreover, a larger sample would have been able to reveal significant differences between the cigarette smokers in both groups if in line with the rates shown in Table 1.

Smoking, hypertension, diabetes or dyslipidemia have been found to be associated with the risk of renal damage in large population studies [15,16] but were omitted from our subsequent analysis as multivariate regression did not reveal any effect.

**Figure 2:**
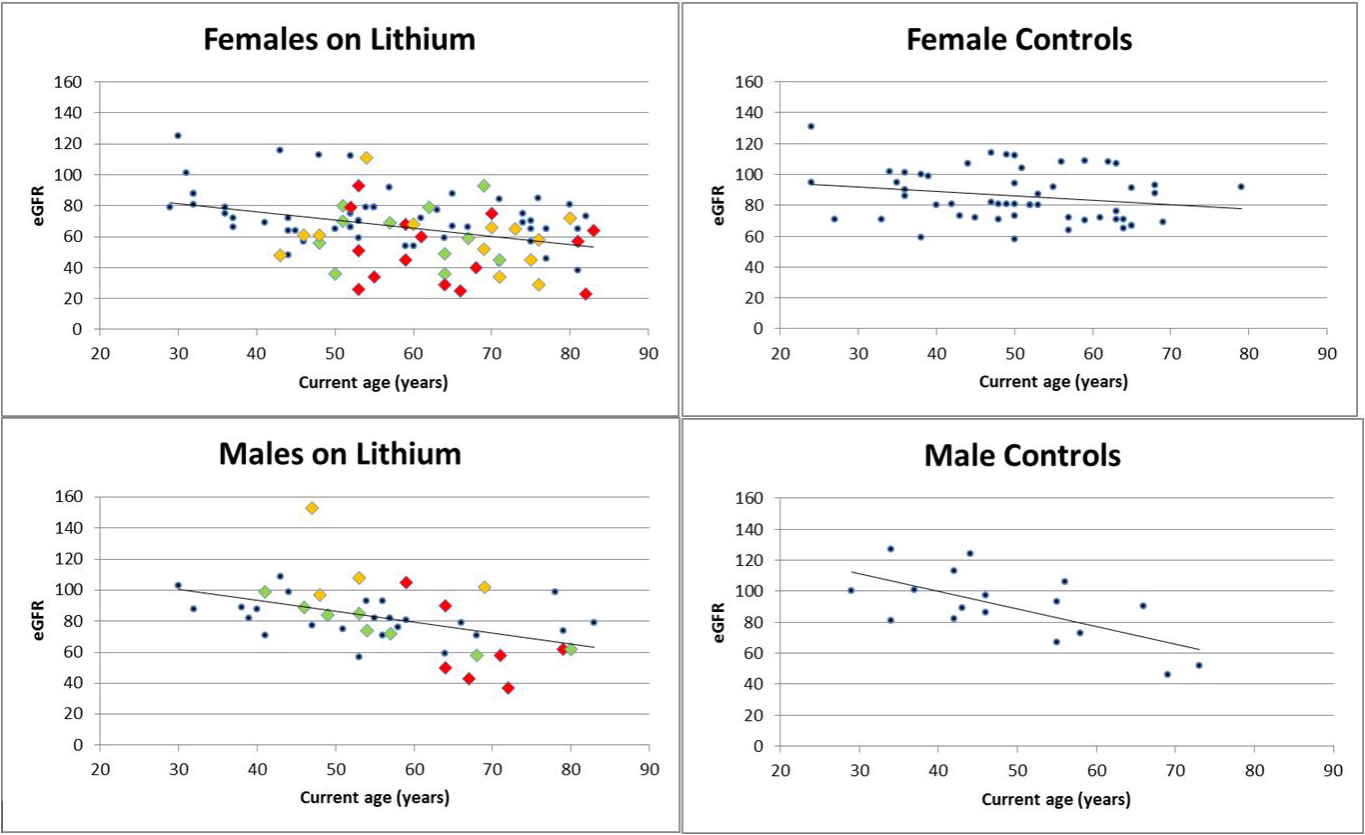
**Distribution of eGFR by age and gender in lithium patients and controls**. Linear regression lines are shown. eGFR = ml/min. Dots = patients with 1 to 15 years of lithium treatment; diamonds = patients with more than 15 years of lithium treatment (green = 16 to 20 years; orange = 21 to 25 years; red = more than 25 years). eGFR, estimated glomerular filtration rate.

On the contrary, the effect of gender and age on eGFR, also known to influence renal function according to population studies [15], was significant. Figure 2 shows the distribution of eGFR by age and gender in lithium patients and controls. Lithium patients, after being checked for age and gender but irrespective of duration of exposure, had significantly lower eGFR (12 ml/min) compared to controls (*P *value = 0.0003; 95% confidence interval (CI) = -17.5 to -6.5 ml/min).

Table 2 shows the results of the multivariate analysis including the duration of lithium treatment. Results can be summarized as follows: eGFR is lower in women (by 11.18 ml/min) and can be estimated to decrease by 0.51 ml/min per year of age and by 0.64 ml/min for each year of lithium treatment. Results were similar if only lithium patients were included (with an eGFR decrease of 0.48 ml/min per year of lithium; *P *= 0.01; 95% CI = -0.85 to -0.11).

**Table 2:** Multivariate analysis of eGFR in lithium patients and controls.

Variable	Beta	*P *value	95% CI	
Current age	-0.51	< 0.001	-0.70	-0.33
Gender (male versus female)	11.18	< 0.001	5.73	16.63
Duration of lithium treatment (years)	-0.64	< 0.001	-0.90	-0.38

Control patients were assumed to have spent zero years on lithium. *F *test = 31.8; *P *value of the model < 0.0000001; *R^2 ^*= 0.32. CI, confidence interval; eGFR, estimated glomerular filtration rate.

## Discussion

Our results, even if limited by the cross-sectional design and relatively small sample size, underscore that the duration of lithium exposure contributes to the effect of advancing age in the decline of renal function. The trend of our results is similar to that reported in a recent paper with a very similar design, that is, a cross-sectional study of 61 patients treated with lithium for a mean of 11.5 years whose eGFR was compared to 62 control patients [17].

With regard to end-stage renal disease, it had long been considered an unlikely event in lithium patients [9] until recently, when Bendz *et al*. suggested that renal failure does occur in chronic lithium treatment, even if it is uncommon [10].

In a systematic review and meta-analysis [4], McKnight and colleagues concluded that the reduction in eGFR in lithium patients is small (a maximum of 5 ml/min/year), representing only 5% of the minimum normal GFR. However, assuming that the decline is linear, 20 years of lithium therapy (a duration which is often attained in prophylaxis) would result in 100% loss of glomerular filtration rate; their estimate, however, was based on prospective studies with a mean observation time of one year on lithium. We maintain that reduced glomerular filtration rate is not clinically negligible even if not falling in CKD stage 5, namely end-stage renal failure, the condition that raised the greatest concern after recent reports [10]. Patients at CKD stage 3 (GFR of 30 to 60 ml/min) raise clinical, ethical and legal questions regarding the decision to continue or discontinue lithium prophylaxis. Moreover, evidence of the decline in renal function with duration of lithium treatment and advancing age may argue for the revision of current guidelines recommending early maintenance in recurrent affective disorder. In our study, we can estimate according to the multivariate analysis, that, for example, all patients 60 years old or older may undergo CKD stage 3 or more severe stages if treated with lithium for 30 years. On the other hand, the incidence of ESRD is considered uncommon based on data from renal dialysis and transplant registers [10]. In any case, the risk of renal failure needs to be weighed against the benefits obtained, as recently reported by Werneke *et al*. [11], who conducted a decision analysis simulating the decision process between physicians and patients, comparing the relative risks and utility of maintenance treatment. The analysis addressed two questions: 'Should lithium be recommended at the beginning of treatment in view of a small but significant risk of ESRD later in life?' and 'Should lithium continuation be recommended even in the presence of long-term adverse renal effects?' This involved weighing the need for effective relapse and suicide prevention from the very beginning of treatment against the risk of lithium-associated ESRD occurring many years later. Indeed, besides its potentially detrimental effects on renal function, lithium has been found to protect not only from recurrence of affective disorders but also from their otherwise high mortality, principally due to suicide [12,18]. In a follow-up of cause-specific mortality in 1,411 patients registered at our lithium clinic between 1980 and 2000, renal failure was recorded as the main cause in two of 201 death certificates, compared with 43 definite suicides (42 of which committed after abandoning lithium prophylaxis) [19]. The progression of renal dysfunction may be very slow, even in patients continuing lithium. In patients at risk of ESRD from other causes, the eGFR point of no return may be set at 30 ml/min. In this cohort, five women fall into this category and are being closely monitored with regard to lithium treatment and renal function. Perhaps it is safe to start early lithium maintenance in patients with normal renal function, provided the function is followed up regularly. Discontinuation of lithium should be considered if the decline in glomerular filtration rate exceeds the expected age-related decline, even though there may be exceptions. The following emblematic case shows the importance of interaction between treating psychiatrists and consultant nephrologists: Belgamwar *et al*. [20] reported a 73-year-old woman with bipolar disorder who was stable on lithium. After long-term use of lithium, she developed chronic renal failure. Doctors decided to stop the lithium and try alternatives, but this proved unsuccessful and resulted in a very poor course, including long hospital admissions. They, therefore, respected the patient's wishes and ability to make a decision and retried lithium. The patient herself and her family accepted this decision despite a high risk of going into dialysis in the future.

We now plan to study the entire cohort of lithium patients registered at our facility over the last three decades (approximately 1,500) to estimate the incidence and time-to-event of the various CKD stages, including patients who had to stop lithium due to very low eGFR or required dialysis or renal transplant. This larger, more comprehensive study should, in part, amend the limitations of this cross-sectional study of 139 lithium patients.

## Conclusions

These preliminary results suggest that reduction in glomerular function, even if rarely progressing to end-stage renal failure, should be reconsidered in the debate on the lithium toxicity profile. Duration of lithium treatment is to be added to advancing age as a risk factor for renal failure. The risk of renal failure must be weighed against the protective effects of lithium on recurrence, quality of life and suicide.

## Abbreviations

CI: confidence interval; CKD: chronic kidney disease; eGFR: estimated glomerular filtration rate; ESRD: end-stage renal disease; GFR: glomerular filtration rate; ICD10: International Classification of Diseases, 10th edition.

## Competing interests

The authors declare that they have no competing interests.

## Authors' contributions

AB conceived of the study, participated in its design and coordination, and drafted the manuscript. RA participated in the design of the study, literature search, data collection, and data interpretation. PC participated in the design of the study, data interpretation, and drafting of the manuscript. FL participated in data collection and data interpretation. CS participated in the design of the study, performed the statistical analysis, and participated in data interpretation. AP performed the assessment of renal function, participated in data interpretation and drafting of the manuscript. MDZ participated in the design of the study and data interpretation. All authors read and approved the final manuscript.

## Authors' information

AB is a Researcher at the Department of Medical Sciences, University of Cagliari and Assistant Medical Director at the Lithium Clinic of the Unit of Clinical Pharmacology, Azienda Ospedaliero-Universitaria di Cagliari, 'San Giovanni di Dio' Hospital, Cagliari. RA is Assistant Medical Director at the Lithium Clinic of the Unit of Clinical Pharmacology, Azienda Ospedaliero-Universitaria di Cagliari, 'San Giovanni di Dio' Hospital, Cagliari. PC is Pharmacist Manager at the Unit of Clinical Pharmacology, Azienda Ospedaliero-Universitaria di Cagliari, 'San Giovanni di Dio' Hospital, Cagliari. FL is a Medical Doctor with a specialization degree in Pharmacology, currently training at the European Medicine Agency, London, UK. CS is a Researcher at the Department of Public Health, University of Cagliari. AP is Chief of the Nephrology, Dialysis and Transplantation Unit, 'G. Brotzu' Hospital, Cagliari, Italy. MDZ is Full Professor of Pharmacology at the Faculty of Medicine, University of Cagliari, Head of the Department of Medical Sciences, University of Cagliari and Chief of the Unit of Clinical Pharmacology, Azienda Ospedaliero-Universitaria di Cagliari, 'San Giovanni di Dio' Hospital, Cagliari.

## Pre-publication history

The pre-publication history for this paper can be accessed here:

http://www.biomedcentral.com/1741-7015/11/33/prepub
